# The Oral–Gut–Immune–Nutrition Axis in Rheumatoid Arthritis: Molecular Mechanisms and Therapeutic Implications

**DOI:** 10.3390/ijms27052385

**Published:** 2026-03-04

**Authors:** Claudia Reytor-González, Náthaly Mercedes Román-Galeano, Lenin Saul Aules-Curicama, Camila Doménica Cevallos-Villacis, Erik González, Dolores Jima Gavilanes, Raquel Horowitz, Daniel Simancas-Racines

**Affiliations:** 1 Facultad de Ciencias de la Salud y Bienestar Humano, Universidad Tecnológica Indoamérica, Ambato 180150, Ecuador; 2 Facultad de Ciencias Médicas, de la Salud y la Vida, Universidad Internacional del Ecuador UIDE, Quito 170411, Ecuador; nathalyroman0001@gmail.com (N.M.R.-G.);; 3 Facultad de Medicina Veterinaria y Zootecnia, Universidad Agraria del Ecuador, Guayaquil 090104, Ecuador; 4 Escuela de Medicina, Universidad Espíritu Santo, Samborondón 0901952, Ecuador; 5 Department of Medicine, Geriatrics Division, Montefiore Medical Center, Bronx, NY 10467, USA

**Keywords:** rheumatoid arthritis, gut–joint axis, oral microbiome, immune dysregulation, dietary modulation, short-chain fatty acids, mucosal immunity

## Abstract

Rheumatoid arthritis is a chronic systemic autoimmune disease that arises from complex interactions among genetic susceptibility, environmental factors, and immune dysregulation. Growing evidence indicates that microorganisms residing in the oral cavity and gastrointestinal tract, together with dietary factors, play a central role in shaping inflammatory and autoimmune responses in rheumatoid arthritis, forming an interconnected microbiome–immune–nutrition axis. Alterations in the composition and function of oral and intestinal microbial communities are associated with disruption of mucosal barrier integrity, activation of innate and adaptive immune pathways, increased differentiation of proinflammatory T lymphocyte subsets, and loss of immune tolerance that promotes autoantibody production. In addition, microbially derived metabolites, particularly short-chain fatty acids, provide a mechanistic link between microbial ecology, immune regulation, and bone metabolism. Diet represents a key upstream modulator of this axis. Dietary patterns rich in anti-inflammatory nutrients support microbial diversity and immunoregulatory metabolite production, whereas diets high in processed foods and saturated fats favor proinflammatory microbial profiles. Accumulating clinical evidence suggests that nutritional strategies and microbiome-targeted dietary interventions may reduce systemic inflammation and disease-related comorbidities when used alongside standard pharmacological treatments. Taken together, the microbiome–immune–nutrition axis represents a modifiable and clinically meaningful target in rheumatoid arthritis, emphasizing the need for interdisciplinary research and well-designed clinical trials to translate these insights into personalized approaches for disease management. The aim of this review is to integrate current mechanistic and clinical evidence on the interactions between the microbiome, immune system, and nutrition in rheumatoid arthritis, with a focus on their pathogenic relevance, therapeutic potential, and implications for personalized, diet-based interventions.

## 1. Introduction

Rheumatoid arthritis (RA) is a chronic, systemic, immune-mediated inflammatory disease characterized by persistent synovitis, autoantibody production, progressive joint destruction, and a wide spectrum of extra-articular manifestations. It affects approximately 0.5–1% of the global population and is associated with substantial morbidity, reduced quality of life, and increased cardiovascular and overall mortality [1,2]. Although RA primarily targets synovial joints, it is now widely recognized as a systemic disease involving complex interactions between genetic susceptibility, environmental exposures, mucosal immunity, and metabolic factors [3].

At the molecular level, RA pathogenesis is driven by a breakdown of immune tolerance, leading to the activation of autoreactive T and B lymphocytes, production of pathogenic autoantibodies (most notably rheumatoid factor (RF) and anti-citrullinated protein antibodies (ACPAs)) and sustained activation of innate immune pathways [4,5]. Proinflammatory cytokines such as tumor necrosis factor (TNF), interleukin (IL)-6, IL-1β, and IL-17 orchestrate synovial inflammation, osteoclast activation, and cartilage degradation [6]. Despite major advances in disease-modifying antirheumatic drugs (DMARDs) and biologic therapies, a substantial proportion of patients exhibit incomplete responses or drug resistance and perceive them as pharmacologically intensive with potential adverse events, underscoring the need to better understand upstream drivers of immune dysregulation in RA [7,8,9].

Over the last decade, the classical joint-centric view of RA has been progressively replaced by a mucosal paradigm of disease initiation [10,11,12]. Accumulating evidence suggests that RA-related autoimmunity may originate at mucosal surfaces, particularly the oral cavity, lungs, and the gastrointestinal tract, well before the onset of clinically apparent arthritis [11]. These sites host dense and metabolically active microbial ecosystems that are in continuous interaction with the host immune system. Perturbations of these ecosystems, commonly referred to as dysbiosis, have been consistently linked to immune-mediated inflammatory diseases, including RA [13].

The oral cavity represents a critical immunological interface, characterized by a unique microbiome shaped by saliva, diet, hygiene, and host immunity [14]. Periodontal disease, a chronic inflammatory condition driven by dysbiotic oral biofilms, is significantly more prevalent and severe in patients with RA compared with the general population [10,12]. Among oral pathogens, *Porphyromonas gingivalis* has received particular attention due to its expression of a unique bacterial peptidylarginine deiminase (PAD), capable of citrullinating host and bacterial proteins [15,16].

Within the periodontal pocket, dysbiotic inflammatory responses are largely driven by specific bacterial toxins that activate the NLRP3 inflammasome [17]. *P. gingivalis* contributes to this process through its lipopolysaccharide (LPS) acting as a potent endotoxin and gingipain exotoxins (RgpA, RgpB, KGP), which directly trigger NLRP3 activation and tissue damage [18]. Concurrently, cytokine activation (particularly IL-1β induced by *Aggregatibacter actinomycetemcomitans*) plays a central role in amplifying local and systemic inflammation. The leukotoxin A (LtxA) expressed by *A. actinomycetemcomitans* activates the NLRP3 inflammasome in leukocytes, triggering a pro-inflammatory form of cell death and promoting IL-1β release [19]. Furthermore, in the presence of bacterial LPS, LtxA facilitates the differentiation of monocyte/macrophage lineage cells into osteoclast-like phenotypes, suggesting a contributory role in osteoclastogenesis by *A. actinomycetemcomitans* and periodontal bone resorption through inflammatory cell-to-cell communication rather than direct cytotoxic mechanisms [19].

**Figure 1:**
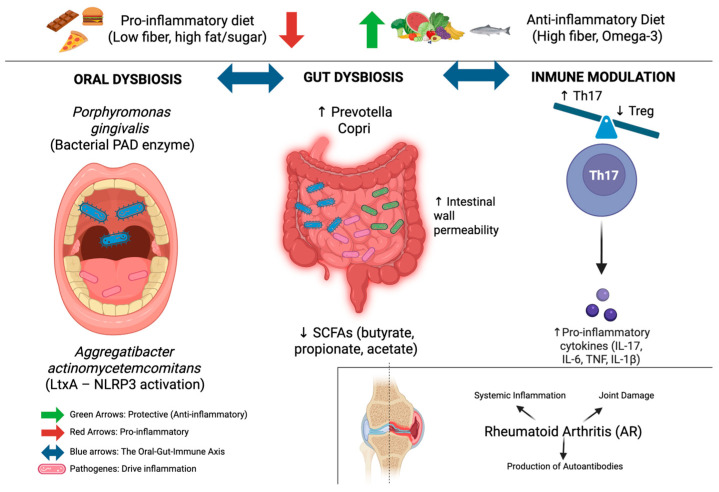
The oral–gut–immune–nutrition axis in RA pathogenesis. Oral dysbiosis (periodontitis) initiates autoantibody formation via *P. gingivalis*-mediated citrullination. This dysbiosis extends to the gut, where microbial imbalances and short-chain fatty acid deficiency compromise barrier integrity, sustaining systemic inflammation and joint damage. Diet acts as a crucial environmental modulator of this entire axis. Abbreviations: ↑, activation/increase; ↓, inhibition/reduction; PAD, peptidylarginine deiminase; LtxA, leukotoxin A; NLRP3, NOD-, LRR- and pyrin domain-containing protein 3; SCFAs, short-chain fatty acids; Th17, T helper 17 cells; Treg, regulatory T cells; IL, interleukin; TNF, tumor necrosis factor; RA, rheumatoid arthritis. Created in https://BioRender.com (accessed on 10 January 2026).

This process may generate neoepitopes that contribute to the breach of immune tolerance and favor ACPA production, thereby providing a mechanistic link between periodontal inflammation and systemic autoimmunity (Figure 1). Nevertheless, this effect is likely mediated indirectly through host protein citrullination and mucosal barrier disruption rather than direct targeting of bacterial autocitrullinated epitopes (in early RA) [10,16,20].

Beyond the oral cavity, gut microbiota has emerged as a central regulator of systemic immune homeostasis. High-throughput sequencing studies have demonstrated distinct alterations in gut microbial composition in patients with early and established RA, including reduced microbial diversity and overrepresentation of proinflammatory taxa such as *P. copri* [21,22,23,24]. These changes are not merely epiphenomena but appear to actively modulate immune responses through microbial metabolites, epithelial barrier function, and antigen presentation pathways [25,26]. Experimental models further support a causal role for gut dysbiosis in arthritis development, showing that specific microbial communities can exacerbate or protect against inflammatory joint disease and modify response to DMARDs [27].

Importantly, the oral and gut microbiomes should not be viewed as isolated compartments, as increasing evidence supports the concept of an oral-gut-immune axis whereby oral microbes and their products can translocate to the gut, influencing intestinal microbial ecology and mucosal immunity [28]. Swallowed oral bacteria, particularly under conditions of periodontal disease, may colonize the gut and amplify systemic inflammatory signaling, reinforcing autoimmune pathways relevant to RA [29,30,31], as illustrated in Figure 1.

Figure 1 Schematic of the oral–gut–immune–nutrition axis in RA.

This axis operates through a complex network of molecular interactions involving innate and adaptive immune pathways, where microbial-associated molecular patterns (MAMPs) (such as LPS, peptidoglycan, and flagellin) engage pattern recognition receptors (PRRs) on epithelial and immune cells [28,32,33]. Aberrant activation of these pathways can skew immune responses toward proinflammatory phenotypes, promoting Th1 and Th17 polarization and suppressing regulatory T cell (Treg) function—hallmarks of RA immunopathology [34,35,36].

Microbial metabolites constitute another critical layer of regulation. Short-chain fatty acids (SCFAs), such as acetate, propionate, and butyrate, produced by gut bacterial fermentation of dietary fiber, exert potent immunomodulatory effects. SCFAs promote Treg differentiation, reinforce epithelial barrier integrity, and suppress inflammatory cytokine production [36,37]. Reduced SCFA-producing bacteria have been reported in RA, suggesting a loss of tolerogenic signals that normally restrain autoimmunity [38].

Nutrition has emerged as a key environmental factor capable of shaping the oral and gut microbiomes while directly modulating immune and inflammatory pathways. Dietary patterns influence microbial composition, metabolic output, and mucosal barrier function, thereby exerting downstream effects on systemic immunity [39,40]. In RA, epidemiological and interventional studies increasingly support a role for diet in disease risk, activity, and therapeutic response [41,42,43].

Western-style diets, characterized by high intake of saturated fats, refined carbohydrates, and ultra-processed foods, have been associated with proinflammatory microbiome profiles, reduced microbial diversity, and impaired gut barrier integrity [44]. These dietary patterns may exacerbate immune dysregulation in genetically susceptible individuals, amplifying RA-related inflammation. In contrast, dietary patterns rich in fiber, polyphenols, omega-3 fatty acids, and micronutrients (such as the Mediterranean diet) have been linked to increased abundance of SCFA-producing bacteria and reduced systemic inflammatory markers in RA patients [45,46,47].

Micronutrients, including vitamin D, zinc, selenium, and iron, further modulate immune function and microbial ecology [48,49]. Vitamin D deficiency, highly prevalent in RA, has been associated with increased disease activity, altered gut microbiota composition, and impaired regulatory immune responses [50,51,52]. Notably, nutrition also affects the oral microbiome, influencing periodontal inflammation and microbial virulence. Diet-induced shifts in oral microbial communities may therefore indirectly contribute to systemic autoimmunity, strengthening the rationale for an integrated oral–gut–immune–nutrition framework in RA [53,54].

Despite rapid advances in microbiome research, immunology, and nutritional science, these domains are often studied in isolation. A growing body of evidence indicates that RA pathogenesis and progression cannot be fully understood without integrating the dynamic interactions between oral and gut microbiota, host immune responses, and nutritional exposures. The concept of an oral–gut–immune–nutrition axis provides a unifying framework to explain how mucosal dysbiosis, metabolic signals, and immune imbalance converge to drive systemic autoimmunity and joint inflammation [55].

The objective of this review is to explore the current evidence, primarily from the last five years, on the molecular mechanisms linking the oral and gut microbiomes with immune dysregulation in RA, with a particular emphasis on the modulatory role of nutrition. To ensure the inclusion of recent mechanistic insights, this narrative review synthesizes peer-reviewed studies published between 2020 and 2026, identified through searches in PubMed, Scopus, and Web of Science using keywords related to the oral–gut–immune–nutrition axis. Foundational studies published before this period were included solely to establish the clinical and epidemiological context of the mucosal paradigm. By integrating findings from human cohort studies, mechanistic experiments, and emerging interventional data, this review aims to provide a comprehensive and clinically relevant perspective on how targeting this axis may inform future preventive and therapeutic strategies in RA. The core findings and themes of this review are summarized in Box 1.

Box 1 **Key Messages of the Narrative Review.**

- Oral-Gut Axis Significance: The oral and gut microbiomes interact to modulate systemic immunity in RA, with oral pathogens potentially colonizing the gut to amplify inflammation.
- Mechanistic Drivers: Periodontitis, particularly driven by *P. gingivalis* and *aggregatibacter actinomycetemcomitans*, triggers citrullination and autoantibody production, while gut dysbiosis reduces Treg and SCFAs.
- Dietary Modulation: High-fiber, anti-inflammatory diets (e.g., Mediterranean) support beneficial SCFA-producing bacteria, whereas Western diets promote proinflammatory dysbiosis.
- Therapeutic Potential: Nutrition-targeted interventions, such as prebiotics and diet modification, are promising adjuncts to standard pharmacological therapies to reduce disease activity and comorbidities.
- Clinical Application: Future RA management requires personalized nutritional strategies based on microbiome profiling to improve treatment responses.

## 2. Microbiome Alterations in Rheumatoid Arthritis

Microbiome alterations are increasingly recognized as disease-modifying features of RA, with measurable shifts in both the oral and intestinal ecosystems across preclinical autoimmunity, early untreated disease, and established RA [56]. Contemporary models position dysbiosis as an “internal environmental” factor that can amplify mucosal inflammation, reshape antigen presentation, and bias adaptive immunity toward autoreactivity, particularly in genetically susceptible hosts [57]. Recent observational studies further suggest that microbiome structure and function may change dynamically around the transition from systemic autoimmunity to clinically apparent synovitis, supporting a role beyond epiphenomenon in at least a subset of patients [58,59].

### 2.1. Oral Dysbiosis in RA: Periodontitis as a Mucosal Amplifier of Autoimmunity

Periodontitis is over-represented in RA populations and is frequently accompanied by an enrichment of periodontal pathobionts, raising the possibility that chronic oral inflammation constitutes a biologically plausible site for the initiation or propagation of RA-related autoimmunity [60,61,62,63]. A central concept is that periodontal dysbiosis is not limited to increased bacterial burden; rather, it reflects a community-level ecological shift that sustains neutrophil-rich inflammation, disrupts epithelial barriers, and promotes persistent antigenic stimulation [29].

Contemporary molecular studies support an association between periodontal microbial signatures and systemic autoantibody profiles. Kim et al. [64], in a 2022 hybrid, translational study, combined human subgingival microbiome profiling and animal models to examine whether periodontal bacteria influence RF induction in RA. Patients with pre-clinical RA showed increased abundance of Porphyromonadaceae and distinct microbial profiles associated with high RF levels, independent of periodontal severity. In collagen-induced arthritis mice, oral inoculation with *P. gingivalis* or *Treponema denticola* similarly worsened arthritis but induced different RF levels, reflecting pathogen-specific effects on humoral immunity. Single-cell RNA sequencing revealed that these differences were linked to distinct B-cell and CD4^+^ Treg activation pathways, supporting a causal, species-dependent role of the periodontal microbiome in RF development during RA pathogenesis [64].

According to a recent systematic review of clinical studies [65], while a polymicrobial dysbiotic community is present in RA patients, *P. gingivalis* and *A. actinomycetemcomitans* remain the most relevant pathogens due to their unique molecular mechanisms for inducing citrullination and driving autoantibody production (ACPA/RF). Other relevant taxa associated with elevated RA disease parameters include *Prevotella_9*, *Peptococcus simiae*, *Aminipila butyrica*, *Leptotrichia* spp., *Neisseria bacilliformis*, *Treponema* sp., and *Parvimonas micra*, which likely contribute to the inflammatory environment [65]

The clinical relevance of periodontopathic exposure is also reflected in therapeutic-response phenotypes. In a 2022 case–control cross-sectional study, markers of infection with periodontal pathogenic bacteria were associated with poorer treatment response, aligning with the concept that persistent oral inflammatory triggers can maintain systemic immune activation and complicate disease control [66]. While causality cannot be asserted from these designs, the consistency of associations across serologic and clinical endpoints reinforces the need to interpret RA as, at least in part, a mucosal immune disorder influenced by chronic microbial dysregulation.

### 2.2. Porphyromonas gingivalis: Mechanistic Plausibility Through Citrullination and Mucosal B Cell Activation

Among oral taxa, *P. gingivalis* has retained particular interest because it expresses a PAD that can citrullinate proteins, potentially increasing the pool of modified antigens relevant to ACPA responses [16]. Although citrullination is a normal post-translational process in humans, RA is characterized by loss of tolerance to citrullinated epitopes [67]. Mechanistically, a dysbiotic periodontal niche enriched in *P. gingivalis* could couple local inflammation (NET formation, protease activity, barrier disruption) with enhanced presentation of modified antigens to mucosal immune cells [68].

Recent evidence strengthens this link by demonstrating antibody responses against citrullinated *P. gingivalis* epitopes in early RA. In 2022, Sherina and colleagues reported increased antibodies to a citrullinated *P. gingivalis* epitope in early RA and provided evidence that gingival tissue B cells can produce such antibodies, supporting a plausible mucosal origin for components of the ACPA response [16]; however, complementary mechanistic work demonstrated that the *P. gingivalis* autocitrullinome itself is not a dominant ACPA target in early RA and that PAD-dependent bacterial citrullination is not required to drive arthritis exacerbation, indicating that periodontal contributions to ACPA may occur indirectly rather than through direct recognition of bacterial autocitrullinated proteins [20]. This observation is relevant because it directly connects the gingival immune compartment, rather than only peripheral blood, to antigen-specific humoral activity, consistent with a model where periodontal inflammation contributes to systemic autoimmunity through local B cell priming and subsequent dissemination or epitope spreading [69].

Beyond PAD, *P. gingivalis* produces gingipains and other virulence factors that can remodel local tissue, alter complement signaling, and intensify inflammatory cascades [70]. Such mechanisms may not be unique to *P. gingivalis*, but the convergence of periodontal niche specialization, immune manipulation, and antigen modification makes this organism a particularly coherent candidate for contributing to RA-relevant immune deviation. The key implication is that “oral dysbiosis” in RA is likely a functional state, defined by inflammatory outputs and immune effects, rather than a single microbe acting in isolation [71].

### 2.3. Aggregatibacter actinomycetemcomitans: Leukotoxin-Driven Hypercitrullination and Risk-State Signals

*A. actinomycetemcomitans* has emerged as a mechanistically distinct periodontal pathogen of interest in RA because LtxA can trigger host neutrophil hypercitrullination by dysregulating endogenous PAD activity, thereby generating citrullinated autoantigens through host enzymes rather than microbial PAD [72]. This proposed pathway is compelling because it integrates hallmark features of RA immunopathology (neutrophil activation, excessive citrullination, and subsequent ACPA generation) within an inflamed periodontal environment. Clinical evidence supporting this mechanism includes a case report where successful antibiotic eradication of a highly leukotoxic *A. actinomycetemcomitans* JP2 strain led to substantial clinical remission of RA symptoms, coinciding with the normalization of ACPA levels [73].

However, contemporary data indicate that the relationship between anti-LtxA responses and RA risk may be population- and context-dependent. In 2023, Martinsson and colleagues assessed antibodies to LtxA in individuals at increased risk of RA and found that serum anti-LtxA was not uniformly elevated before diagnosis, and that associations with progression and ACPA levels differed between populations [74]. In line with this variability, evidence from A clinical cohort of 115 patients suggests that *A. actinomycetemcomitans* abundance is more consistently associated with established RA disease activity and periodontal inflammation than with uniform preclinical serological responses [75]. These findings do not negate mechanistic plausibility; rather, they suggest heterogeneity in the relevance of *A. actinomycetemcomitans*-linked pathways across cohorts and emphasize the need for integrated approaches that consider broader oral community structure, host genetics, smoking, periodontal severity, and mucosal immune phenotypes.

From a translational standpoint, the *A. actinomycetemcomitans* story underscores a recurrent theme in RA microbiome research: microbial contributions may be conditional (restricted to subgroups), temporally specific (most relevant in risk states or early disease), or mediated by discrete host responses (e.g., neutrophil PAD activation) rather than by stable colonization alone [65,76,77]. Furthermore, recent evidence indicates that systemic neutralizing antibodies against LtxA do not fully reflect the actual presence of *A. actinomycetemcomitans* in the saliva, suggesting that local colonization does not always induce a detectable systemic serological response [78].

### 2.4. Gut Dysbiosis in RA: Reduced Diversity and Functional Remodeling of Mucosal Immunity

Regarding the link between oral microbes and the gut, it is increasingly recognized that oral bacteria can survive the gastric passage and colonize the intestinal tract, a phenomenon sometimes referred to as the ‘oral-gut axis’ [79]. We speculate that in RA, reduced gastric acidity (potentially due to medication or autoimmunity) combined with periodontal biofilm disruption may enhance the translocation of pathobionts like *P. gingivalis* or *A. actinomycetemcomitans* to the intestine. Once in the gut, these oral microbes may directly exacerbate dysbiosis, disrupt the mucosal barrier, and trigger further pro-inflammatory responses, thereby linking periodontal inflammation directly to systemic autoimmunity [80,81].

In parallel with oral findings, gut dysbiosis in RA is commonly characterized by reduced microbial diversity and compositional shifts that collectively favor pro-inflammatory immune programming [82]. Large-scale syntheses published within the past five years support that gut microbial alterations are not unique to RA but are prominent across rheumatic diseases, with RA showing reproducible patterns in multiple cohorts. A 2022 systematic review and meta-analysis of 92 observational studies across rheumatic diseases reported consistent gut dysbiosis signals, reinforcing that altered intestinal ecology is a frequent correlate of systemic inflammation and autoimmunity [83]. While such meta-analytic approaches face heterogeneity in sampling, sequencing platforms, geography, diet, and medication exposures, they strengthen confidence that RA-associated dysbiosis is not solely a single-study artifact.

More granular clinical cohort studies add details regarding diversity and clinical correlates. In a 2023 cohort of established RA, gut microbial composition differed from controls, and younger RA patients demonstrated reduced richness and evenness; specific microbial signatures also showed potential value in predicting response to second-line conventional synthetic DMARDs [21]. This is important because it indicates that RA dysbiosis can persist beyond the untreated early phase and may intersect with therapeutic outcomes, supporting bidirectional relationships among inflammation, medications, and microbial metabolism.

Longitudinal work in individuals at risk of RA provides additional evidence that gut microbial communities may change around disease transition. Rooney and colleagues (2024) reported structural, functional, and temporal microbiome differences between progressors and non-progressors, addressing prior inconsistencies about Prevotellaceae abundance and highlighting microbiome instability as a potential feature in the lead-up to clinical onset [58]. Such studies are especially informative because they minimize reverse causation linked to long-standing inflammation and prolonged immunomodulatory therapy, though they still require careful control of antibiotics, diet, and other confounders.

A 2024 systematic review and meta-analysis by Su and colleagues further reinforces these observations by quantitatively demonstrating a significant reduction in gut microbial α-diversity in RA compared with healthy controls. Importantly, the decrease in microbial richness and evenness was most pronounced in treatment-naïve patients, whereas individuals receiving antirheumatic therapy showed less marked alterations, supporting the concept that dysbiosis is closely linked to early disease biology rather than solely to inflammatory burden or chronic treatment effects. Consistent with broader rheumatic disease analyses, no clear association was observed between α-diversity and baseline disease activity, suggesting that altered gut ecology represents a disease-associated trait rather than a direct marker of clinical severity [84].

However, it is crucial to recognize that RA treatments substantially modulate the gut microbiota. For instance, DMARDs like methotrexate have been shown to alter microbial composition, while biological agents, particularly TNF inhibitors, may partially restore a healthier microbiome profile by reducing systemic inflammation [85,86].

### 2.5. Enrichment of Prevotella copri: From Association to Immune-Relevant Function

Enrichment of *P. copri* (now often classified as *Segatella copri* in updated taxonomy) [87] has been repeatedly reported in early or untreated RA and in at-risk states, although the strength and direction of association can vary by geography, diet, and baseline Prevotellaceae prevalence [88,89]. Contemporary reviews emphasize that *P. copri* signals are most consistent in new-onset or preclinical contexts rather than in long-treated established disease, implying a time-sensitive contribution.

Mechanistically, *P. copri* has been linked to Th17-skewing immune responses and mucosal inflammation, plausibly through antigenic stimulation, effects on epithelial barrier integrity, and remodeling of microbial metabolite pools. While earlier experimental work established proof-of-concept for Prevotella-driven immune deviation in arthritis-prone models, recent human observational and longitudinal data better define when and in whom these signals emerge [59,88]. The key clinical inference is that Prevotellaceae enrichment may represent a “window” biomarker of imminent transition in a subset of at-risk individuals, rather than a universal diagnostic feature across all RA stages.

### 2.6. Depletion of SCFA-Producing Bacteria: Loss of Regulatory Metabolites and Barrier Support

A frequently described functional hallmark of RA-associated gut dysbiosis is the depletion of SCFA-producing bacteria, including taxa that generate butyrate and related metabolites that support epithelial barrier integrity and promote immunoregulatory pathways (e.g., Treg cell differentiation and anti-inflammatory signaling) [38]. Although SCFAs act locally in the gut, they also enter the systemic circulation, where they exert anti-inflammatory effects on peripheral immune cells. Consequently, the depletion of SCFA-producing bacteria in RA results in lower serum concentrations of these metabolites, thereby reducing their capacity to regulate systemic inflammation and potentially failing to inhibit synovial inflammation [90,91]. Loss of these functions can increase intestinal permeability and facilitate translocation of microbial products (e.g., LPS), enhancing systemic innate immune activation and potentially amplifying synovial inflammation.

Recent mechanistic syntheses describe multiple convergent pathways by which gut dysbiosis can promote arthritis, including altered barrier function, microbial metabolites, molecular mimicry, and reshaped innate-adaptive crosstalk [92]. In established RA cohorts, intestinal community shifts have also been associated with immune parameters such as CD4+ Treg subpopulations and cytokine profiles, supporting the concept that compositional changes can translate into measurable immunologic remodeling [93]. While these association studies do not prove causality, they align with a biologically coherent framework in which reduced SCFA production and impaired mucosal tolerance contribute to systemic inflammatory setpoints.

Notably, dysbiosis signatures may be more pronounced in difficult-to-treat RA phenotypes. In 2025, Ruiz-Limón and colleagues reported gut microbiota differences and inflammatory associations in difficult-to-treat RA compared with easier-to-treat disease, supporting the possibility that persistent microbial dysfunction contributes to refractory inflammation in some patients [94]. This has direct therapeutic implications, as it suggests that microbiome-targeted strategies may be most relevant in defined clinical subgroups rather than as universal adjuncts.

### 2.7. Integrating Oral and Gut Dysbiosis: Shared Immune Pathways and Stage-Specific Signals

Although the oral and gut microbiomes are distinct ecosystems, they may converge on shared immune mechanisms relevant to RA: chronic neutrophil activation, excessive citrullination (via microbial or host PAD pathways), epithelial barrier disruption, and sustained antigen presentation that biases adaptive immunity toward autoreactivity [24,28]. Oral dysbiosis may be particularly relevant for local citrullinated antigen generation and mucosal B cell activation (as supported by gingival B cell antibody production) [16,71,76], whereas gut dysbiosis may more strongly influence systemic immune tone through metabolite depletion (including SCFAs), barrier permeability, and Th17/Treg imbalance [92,93].

Across both sites, an emerging pattern is temporality and heterogeneity: certain microbes or signatures appear most prominent in preclinical autoimmunity or early untreated RA [16,58,59], while others persist in established disease and relate to treatment response or refractory phenotypes [21,94]. These observations argue against a single “RA microbiome” and favor a stratified model in which dysbiosis patterns map onto disease stages, immune endotypes (ACPA-positive vs. ACPA-negative), environmental exposures, and medication histories.

## 3. Microbiome–Immunity Interactions in Rheumatoid Arthritis

### 3.1. Mucosal Immune Activation and the Th17-Skewed Inflammatory Set Point

The oral cavity and gastrointestinal tract provide dense antigenic stimulation, continuous innate sensing, and metabolic signaling, all of which can bias adaptive responses toward pathogenic inflammation in RA. Across cohorts, RA and “pre-RA” states (e.g., ACPA-positive individuals without clinical arthritis) show microbiome and metabolome signatures consistent with impaired barrier integrity, heightened innate activation, and expansion of effector T-cell programs, especially Th17 polarization, alongside inadequate counter-regulation by FoxP3+ Treg and follicular Treg (Tfr). These pathways provide a mechanistic bridge between dysbiosis and hallmark RA cytokine networks (TNF, IL-6, IL-1β, GM-CSF, IL-17A/F) and help explain how mucosal perturbations can be translated into systemic autoimmunity and joint inflammation [38,95,96,97,98].

At the mucosa, dysbiosis can increase exposure to MAMPs such as LPS, peptidoglycan fragments, and flagellin [32]. These ligands engage pattern-recognition receptors (including Toll-like receptors and NOD-like receptors) on epithelial cells, dendritic cells, macrophages, and innate lymphoid cells, promoting production of IL-1β, IL-6, IL-23, and chemokines that support Th17 differentiation and recruitment [22,99,100]. In parallel, RA-associated barrier disruption (“leaky gut”) can facilitate translocation of microbial products and metabolites into circulation, amplifying systemic innate activation and priming peripheral lymphoid compartments [101,102]. Although RA therapies targeting TNF and IL-6 can suppress downstream inflammation, upstream mucosal drivers may persist, potentially contributing to disease initiation, flare biology, and variability in therapeutic response.

### 3.2. Th17 Activation, Cytokine Amplification, and Loss of Immune Tolerance

Th17 cells are central to mucosal defense but become pathogenic when differentiation and effector programs are sustained by chronic antigenic stimulation and inflammatory cytokines [103]. In RA, Th17-associated cytokines promote synovial inflammation, angiogenesis, osteoclastogenesis, and cartilage destruction. Mechanistically, dysbiosis can reinforce the IL-23/IL-17 axis through multiple convergent inputs: antigen presentation of microbial or modified self-peptides; dendritic-cell production of IL-1β, IL-6, and IL-23; and metabolic cues that favor Th17 differentiation over Treg stability [104]. The resulting cytokine milieu can then “lock in” inflammatory loops within synovial tissue, where fibroblast-like synoviocytes, macrophages, and infiltrating lymphocytes reciprocally sustain TNF, IL-6, IL-1β, and IL-17 signaling [6].

A complementary feature is failure of immune tolerance. Beyond classical FoxP3+ Treg impairment, recent evidence highlights a specific defect in Tfr-mediated control of germinal center reactions in RA, linking dysbiosis and metabolite alterations to exaggerated humoral autoimmunity [35,105]. In an integrative analysis connecting gut microbial patterns, circulating metabolites, and immune phenotypes, reduced Tfr cells were associated with impaired immune tolerance in RA, consistent with a permissive environment for autoreactive B-cell selection and autoantibody maturation [106]. This is clinically relevant because germinal center dysregulation supports epitope spreading and persistence of high-affinity ACPAs, which strongly associate with erosive disease and extra-articular manifestations.

### 3.3. Microbial Metabolites as Immune Regulators: SCFAs and Beyond

The microbiome’s functional output (particularly its metabolites) often provides the most direct mechanistic pathway from community structure to immune tone [107]. SCFAs such as acetate, propionate, and butyrate arise largely from microbial fermentation of dietary fibers [108]. SCFAs influence epithelial integrity, innate immune activation, antigen presentation, and adaptive differentiation through G-protein coupled receptors (e.g., FFAR2/GPR43, FFAR3/GPR41) and epigenetic effects (notably histone deacetylase inhibition by butyrate) [109]. In RA, multiple lines of evidence converge on reduced SCFA availability and impaired SCFA-linked immune regulation. In a 2020 study, RA patients and arthritic mice showed reduced microbial-derived SCFAs compared with controls; in mice, butyrate supplementation reduced arthritis severity in a Breg- dependent manner by enhancing an AhR-linked transcriptional program that supports IL-10 competent Breg function and constrains germinal center/plasmablast differentiation [98].

Human longitudinal data strengthen the clinical plausibility of SCFAs as tolerance-promoting mediators in the transition from autoimmunity to arthritis. In ACPA-positive individuals at increased risk for RA, higher serum SCFA levels were associated with non-progression to inflammatory arthritis, suggesting that preserved SCFA signaling may help maintain immune quiescence despite established autoimmunity [110]. Findings support a model in which SCFAs bolster barrier function and restrain inflammatory differentiation pathways (including Th17 programs) while reinforcing regulatory circuits (Bregs, Tregs, and possibly Tfr cells). Consistent with this, experimental and translational work continues to explore how augmenting butyrate delivery can suppress autoimmune arthritis, including pharmacologic approaches designed to improve oral bioavailability of butyrate and enhance systemic immunomodulatory impact [111].

Propionate and butyrate can also shape B-cell fate decisions relevant to autoantibody production. In a study examining SCFA signaling through FFAR2, SCFAs regulated B-cell differentiation and alleviated arthritis in experimental models, supporting the idea that microbial metabolites influence RA not only through T-cell polarization but also through germinal center biology and antibody effector pathways [112]. These effects are conceptually aligned with observations that impaired Tfr-mediated control is linked to altered gut microbiota and metabolite profiles in RA [106].

### 3.4. Succinate as a Pro-Inflammatory Metabolic Signal in RA

While SCFAs are often immunoregulatory, other microbiome-linked metabolites can be pro-inflammatory in specific contexts. Succinate is a prominent example: it can function as both a metabolic intermediate and a signaling molecule via SUCNR1 (GPR91) [113]. Succinate accumulation is associated with inflammatory macrophage programs and can amplify IL-1β production through HIF-1α-related pathways; RA synovial fluid has been reported to contain abundant succinate, positioning it as a candidate mediator linking inflammation and metabolism within joints [114]. Endothelial and myeloid responses to succinate can facilitate leukocyte recruitment, vascular activation, and cytokine amplification, thereby reinforcing synovial inflammation. A focused synthesis of the succinate/IL-1β axis emphasizes that succinate can act upstream of IL-1β in RA synovial fluid and can promote inflammatory phenotypes that are relevant to tissue damage [114].

From a microbiome-immunity perspective, succinate is also influenced by microbial ecology: certain dysbiotic configurations can increase succinate production or decrease its consumption, contributing to altered luminal and systemic metabolite pools [115]. In RA, a pro-succinate environment may complement Th17-skewing signals, because IL-1β and IL-6 support Th17 differentiation and expansion [116]. Thus, succinate may operate as a metabolic amplifier that integrates microbial functional outputs with cytokine cascades central to RA pathobiology [117,118].

### 3.5. ACPAs, Germinal Center Biology, and Mucosal Triggers

ACPAs are a defining feature of seropositive RA and can predate clinical arthritis by years, indicating that ACPA generation is often an early immunologic event [119]. This process is initiated at mucosal interfaces, with the oral mucosa serving as a critical priming site where chronic periodontal inflammation and specific pathobionts facilitate protein citrullination and autoreactive B-cell activation [120]. Mucosal sites likely contribute to this process via (i) increased protein citrullination driven by inflammation and microbial enzymes, (ii) presentation of citrullinated self-antigens and modified microbial proteins, and (iii) expansion of autoreactive B-cell clones in permissive germinal center environments [121]. Dysbiosis-associated defects in regulatory pathways (particularly those involving Bregs and Tfr cells) provide a mechanistic rationale for how autoantibody maturation may escape normal checkpoints. In the 2020 study noted above, butyrate supplementation constrained germinal center/plasmablast differentiation via an AhR-dependent program supporting Breg function, directly connecting microbial metabolites to humoral autoimmunity control [98]. Separately, reduced Tfr cells linked to dysbiosis and altered metabolites in RA offers another pathway through which microbiome changes could increase the likelihood of sustained autoantibody responses [106].

Microbial community members can also promote preclinical autoimmunity in experimental systems. For example, expansion of *Eggerthella lenta* augmented preclinical RF production in mice and increased disease severity when arthritis was induced, with immunologic features including increased CD4 T-cell responses and cytokine-producing B-cell subsets [122]. Although RF is distinct from ACPA, these data reinforce the concept that specific taxa can shift immune set points toward autoantibody-prone states, potentially interacting with host genetics and mucosal inflammation to shape RA trajectories.

### 3.6. Molecular Mimicry: Linking Microbial Antigens to Autoreactive T- and B-Cell Responses

Molecular mimicry refers to immune cross-reactivity driven by structural similarity between microbial epitopes and self-antigens, enabling microbial exposures to activate autoreactive lymphocytes [123]. In RA, mimicry has long been hypothesized, but recent work has strengthened experimental support by demonstrating HLA-restricted T-cell activation by bacterial peptides resembling joint-relevant epitopes. In a study focusing on the RA-associated HLA-DRB1*04:01 context, a bacterial L-asparaginase-derived peptide (L-ASNase67-81) showed strong predicted binding and structural mimicry of an immunodominant type II collagen epitope, and it activated CD4+ Treg with increased production of IL-2, IL-17A/F, and IFN-γ in early RA samples [124]. This provides a contemporary mechanistic example of how microbial proteins can engage disease-relevant antigen presentation and promote Th17-associated cytokine outputs.

Mimicry and post-translational modification may also intersect in RA because citrullination can alter peptide binding to shared-epitope HLA class II alleles and enhance immunogenicity. Therefore, mimicry is likely not a single-pathogen phenomenon but a network property: repeated exposures to distinct microbial antigens, combined with inflammation-driven citrullination and permissive germinal center responses, can broaden autoreactivity over time. Recent reviews emphasize this multifactorial view and integrate mimicry with other mechanisms (barrier dysfunction, metabolite shifts, and innate immune amplification) to explain why no single organism consistently accounts for RA initiation across populations [12,125].

## 4. Nutrition as a Modulator of the Microbiome–Immune Axis

### 4.1. Diet as an Upstream Determinant of Dysbiosis and Immune Tone in RA

RA is increasingly framed as a systemic immune disease shaped by mucosal interfaces, where diet influences both microbial ecology and host immunometabolism. Dietary exposures regulate substrate availability for microbial fermentation, bile acid pools, epithelial barrier integrity, and the balance of tolerogenic versus proinflammatory immune programs (notably Th17/Treg dynamics). Contemporary RA literature supports a bidirectional model in which chronic inflammation and immunomodulatory therapies alter the gut environment, while diet-driven microbial shifts and metabolite signaling can amplify or dampen synovial inflammation through the gut-immune-joint axis. Recent integrative reviews emphasize that nutrition is one of the most actionable levers to reshape microbiota composition and function, with plausible downstream effects on cytokine networks, innate immune activation, and adaptive autoimmunity [126,127,128].

In RA, dysbiosis has been characterized by reduced microbial diversity and depletion of SCFA, producing taxa, alongside expansion of pathobiont-associated signatures in susceptible phenotypes [24,127]. These changes have functional consequences: SCFAs (butyrate, propionate) support epithelial integrity and regulatory immune signaling, while alternative metabolite profiles (including succinate and bile-acid derivatives) may promote inflammatory myeloid activation and Th17 polarization [129]. Diet patterns that increase fermentable fibers and polyphenol-rich plant foods tend to favor SCFA producers and anti-inflammatory metabolite profiles [130], whereas Western dietary patterns (high saturated fat, refined carbohydrates, ultra-processed foods) tend to reduce microbial diversity, increase endotoxin burden, and bias toward barrier disruption and low-grade systemic inflammation [131,132].

### 4.2. Anti-Inflammatory Dietary Patterns in RA: Mediterranean and Plant-Forward Approaches

#### 4.2.1. Mediterranean Dietary Pattern

The Mediterranean diet (MedDiet) is the most consistently discussed “anti-inflammatory” pattern in RA because it combines high intake of plant foods (vegetables, legumes, fruits, nuts), extra-virgin olive oil, and fish, elements linked to higher microbial diversity, greater SCFA production capacity, and reduced proinflammatory lipid signaling [133,134,135]. Contemporary clinical literature suggests that MedDiet adherence can improve patient-reported outcomes and functional measures in managing RA [45,136,137]. A randomized controlled trial of a Mediterranean dietary intervention in RA has been published recently, designed around clinical endpoints such as physical function and quality of life, reflecting renewed interest in structured dietary therapy as an adjunct to standard pharmacologic care [138,139].

#### 4.2.2. Plant-Based and Plant-Forward Strategies

Plant-forward dietary strategies (ranging from vegetarian/vegan diets to predominantly plant-based anti-inflammatory patterns) are increasingly studied in inflammatory conditions because they raise fiber and polyphenol intake while lowering saturated fat and advanced glycation end products [140]. In RA, recent clinical work supports the feasibility of plant-forward strategies and signals benefits in disease activity and metabolic endpoints when implemented in structured formats, including protocols that use short-term fasting as a metabolic “reset” before transitioning to plant-based maintenance. These approaches are hypothesized to reduce inflammatory signaling via multiple convergent pathways: (i) enrichment of SCFA-producing taxa through higher fiber intake; (ii) reduced bile acid and endotoxin-driven innate immune activation due to lower saturated fat; and (iii) polyphenol-mediated modulation of microbial composition and host inflammatory gene expression [141]. Importantly, the clinical impact of dietary patterns in RA is shaped by baseline therapy, disease duration, adherence, and comorbidities (obesity, insulin resistance) [142]. Observational nutrition studies in RA continue to show associations between overall dietary quality and inflammation/disease activity markers, supporting the concept that diet is relevant even in pharmacologically treated cohorts [143].

### 4.3. Fermentable Fiber as a Driver of SCFA Production and Immunoregulation

#### 4.3.1. Fiber-Microbiome-SCFA Pathway

Fermentable fibers (inulin-type fructans, resistant starches, pectins, beta-glucans) are central to immunologically relevant microbiome modulation because they are metabolized into SCFAs, principally acetate, propionate, and butyrate. SCFAs modulate immune responses by acting as signaling molecules through G-protein-coupled receptors (e.g., GPR41/43/109A) on immune cells and by promoting epithelial barrier integrity through the bolstering of tight junction proteins like zonulin [144,145]. Epigenetically, butyrate acts as a histone deacetylase (HDAC) inhibitor in intestinal epithelial and dendritic cells, promoting the transcription of anti-inflammatory genes [146]. This reinforcement reduces the translocation of pathobiont-derived products, such as *A. actinomycetemcomitans* or *P. gingivalis* LPS, thereby curbing the downstream activation of the TLR4/NF-κB pathway and subsequent IL-6 and TNF-*α* production [147,148]. In autoimmune models and translational immunology, butyrate is repeatedly linked to Treg induction and suppression of excessive Th17 responses, supporting a mechanistic rationale for fiber-based interventions in RA [149].

#### 4.3.2. Human Interventional Evidence in RA

Recent RA trials evaluating fiber or prebiotic supplementation have expanded beyond general dietary counseling. A 2025 randomized, triple-blind clinical trial reported that 10 g/day inulin for 8 weeks improved multiple clinical outcomes and inflammatory indices in RA, including improvements in morning stiffness and biomarkers such as CRP in the intervention group [150]. This trial is notable because it directly tests a fermentable substrate with a known microbial fermentation profile, aligning clinical outcomes with a plausible microbiome-mediated mechanism.

Additionally, a randomized clinical trial of high-fiber multigrain supplementation in RA (12 weeks) reported improvements in disease activity scores and inflammatory/oxidative stress biomarkers, supporting the concept that complex fiber matrices can yield clinically meaningful effects when adherence is adequate [151]. A separate double-blind randomized intervention incorporating medium-chain triglycerides (MCT) with and without fiber reported effects on disease activity endpoints in RA, reflecting growing interest in combining lipid and fiber modulation to shape both microbiome outputs and host immunometabolism [152].

Not all evidence is interventional; population-level analyses also support relevance. For example, higher dietary fiber intake has been associated with lower RA prevalence and lower inflammatory indicators in nationally representative datasets, consistent with a protective signal for fiber-rich dietary patterns [153]. While cross-sectional designs cannot establish causality, these findings are coherent with interventional data and mechanistic immunology.

#### 4.3.3. Polyphenols, Microbiome Remodeling, and Inflammatory Signaling in RA

Polyphenols represent a major diet-derived class of bioactives that interact with the microbiome bidirectionally: gut microbes metabolize polyphenols into bioactive derivatives, while polyphenols selectively inhibit or promote taxa, often favoring beneficial commensals and SCFA producers. In RA, polyphenols are relevant because they can reduce NF-κB signaling, oxidative stress pathways, and NLRP3 inflammasome activation, while potentially shifting the cytokine milieu away from IL-6/TNF/IL-17-dominant profiles [130].

Mechanistically, specific polyphenols such as curcumin directly bind to and inhibit the NLRP3 inflammasome complex, reducing the maturation and secretion of IL-1 β in macrophages stimulated by oral pathobionts [154]. Furthermore, they enhance the expression of Nrf2, triggering an antioxidant response that protects the gut mucosa from oxidative stress-induced damage [155].

Recent evidence syntheses in RA have strengthened the clinical signal for polyphenol interventions. A 2023 systematic review and meta-analysis of randomized controlled trials concluded that dietary polyphenols improved RA-related outcomes across multiple endpoints, albeit with heterogeneity in formulations, dosing, and study quality [156]. This is complemented by contemporary curcumin-focused evidence syntheses (systematic review/meta-analysis), which suggest improvements in inflammation and symptom measures in RA cohorts, while emphasizing variability in bioavailability and trial designs [157].

From a microbiome perspective, polyphenols may complement fiber by enhancing ecological stability and metabolic capacity for anti-inflammatory metabolites. This matters in RA because dysbiosis frequently includes loss of taxa that support barrier integrity and immunologic tolerance [24,127]. Although more RA trials that include paired microbiome-metabolome endpoints are needed, the convergent evidence supports polyphenols as a plausible adjunct strategy to modulate the microbiome-immune axis alongside conventional disease-modifying therapy.

#### 4.3.4. Omega-3 Fatty Acids: Immunometabolic Modulation and Microbiome-Linked Effects

Omega-3 polyunsaturated fatty acids (PUFAs) (EPA, DHA) have long been studied in RA due to their capacity to generate specialized pro-resolving mediators (resolvins, protectins, maresins) and to compete with arachidonic acid for eicosanoid synthesis, thereby reducing proinflammatory lipid mediator production.

A 2024 systematic review/meta-analysis of randomized placebo-controlled trials reported that omega-3 supplementation had small-to-modest effects on several disease activity indicators, with overall certainty of evidence often rated low due to heterogeneity and trial limitations [158]. Another 2024 meta-analysis focusing on lipid metabolism and clinical activity reported improvements in fatty acid profiles and reductions in tender joint count, with smaller or non-significant effects on global indices such as DAS28 in pooled analyses [159]. These findings remain clinically relevant because omega-3s are generally safe, may improve cardiometabolic risk profiles, and could provide incremental benefit as an adjunct, particularly in patients with low baseline omega-3 intake or high omega-6:omega-3 ratios.

Emerging work suggests omega-3s may also influence gut microbial composition and permeability through bile acid signaling and anti-inflammatory effects at the mucosal surface, but RA-specific microbiome-linked omega-3 trials remain limited [160]. Therefore, omega-3s are best positioned as an immunometabolic adjunct with potential microbiome interactions rather than a primary microbiome-targeting therapy.

#### 4.3.5. Vitamin D: Immune Tolerance, Barrier Function, and Potential Microbiome Relevance

Vitamin D has immunomodulatory effects that are mechanistically aligned with RA pathobiology: it may inhibit Th17 differentiation, support Treg function, and modulate antigen-presenting cell activation [161]. Vitamin D receptors are expressed widely in immune and epithelial tissues, and vitamin D signaling can influence barrier integrity and antimicrobial peptide expression, features relevant to microbiome stability and endotoxin translocation.

Recent evidence syntheses and trials continue to evaluate whether supplementation translates into clinically meaningful RA improvements. A 2024 systematic review and meta-analysis of randomized clinical trials assessed vitamin D supplementation effects on inflammatory and clinical outcomes in RA, reflecting ongoing uncertainty about effect size, optimal dosing, and patient selection [162]. Contemporary randomized controlled evidence (2024–2025) includes controlled trials assessing disease activity and fatigue/pain outcomes, indicating that vitamin D supplementation may improve some measures in selected cohorts; however, results vary across studies, and baseline deficiency status appears important [163,164].

From the microbiome perspective, vitamin D deficiency has been linked to altered gut microbiome profiles in other musculoskeletal inflammatory conditions, and RA-focused reviews now frequently discuss vitamin D as a factor that may influence microbiome composition and immune responses; however, direct RA microbiome-interventional data remain limited [165]. In clinical practice, the most defensible approach is targeted correction of deficiency with monitoring, recognizing that benefits may be mediated through immune and barrier effects even if microbiome changes are not directly measured.

#### 4.3.6. Zinc and Micronutrient Status: Immunologic Competence and RA-Relevant Outcomes

Zinc is essential for innate and adaptive immune function, epithelial barrier maintenance, and antioxidant defense. In RA, micronutrient considerations are clinically relevant because chronic inflammation, altered dietary intake, and corticosteroid use can influence nutritional status, while immune activation increases metabolic demand for micronutrient-dependent pathways [166].

Recent evidence indicates that zinc status is frequently altered in RA. Fang and colleagues (2024) examined the association between dietary zinc intake and the prevalence of osteopenia or osteoporosis in patients with RA using nationally representative data from the NHANES cohorts (2007–2010, 2013–2014, and 2017–2020). In a cross-sectional analysis of 905 RA patients aged 40 years and older, weighted univariate and multivariate logistic regression models demonstrated that higher dietary zinc intake was independently associated with significantly lower odds of osteopenia or osteoporosis after adjustment for relevant covariates. This protective association was particularly evident in subgroups including older adults (≥60 years), individuals with normal or low BMI, NSAID users, and patients with dyslipidemia, diabetes, or hypertension. The findings suggest that adequate zinc intake may confer bone-protective benefits in RA, although the authors emphasize the need for longitudinal and interventional studies to confirm causality and elucidate underlying mechanisms [167].

However, robust clinical trials of zinc supplementation specifically targeting RA disease activity and microbiome outcomes remain sparse, and much of the experimental zinc literature is preclinical or focused on immune/oxidative endpoints rather than microbiome-resolved mechanisms [168]. For clinical translation, zinc should be framed as a supportive factor, preferably optimized through diet and corrected when deficient, rather than a standalone RA anti-inflammatory intervention.

#### 4.3.7. Western Dietary Pattern and Pro-Inflammatory Dysbiosis in RA

Western dietary patterns are associated with microbial configurations that favor dysbiosis, reduced microbial diversity, and increased intestinal permeability, promoting endotoxin exposure and chronic low-grade inflammation [44]. General nutrition-microbiome evidence supports a consistent association between Western patterns and gut dysbiosis with inflammatory consequences, which is highly relevant to RA because innate immune activation and mucosal barrier disruption can amplify systemic cytokine signaling [131].

In the RA-focused literature, recent reviews explicitly highlight diet-driven dysbiosis as a modifiable contributor to the gut-joint axis, linking Western dietary features to unfavorable microbial and immune profiles [57]. Observational studies using metrics such as the Dietary Inflammatory Index (DII) have also reported associations between pro-inflammatory dietary patterns and RA activity, providing a quantitative bridge between dietary pattern exposure and clinical phenotype [169]. Although such studies cannot establish causality, they strengthen the rationale for dietary interventions that reduce inflammatory dietary load and support microbial resilience.

At a molecular level, Western diets may promote bile acid profiles and microbial metabolites that support inflammatory signaling, increase oxidative stress, and bias T-cell responses toward Th17 polarization, while reducing SCFA availability that normally supports epithelial integrity and Treg-mediated tolerance. This mechanistic framing is consistent with emerging models of RA as a disease in which mucosal immune activation contributes to systemic autoimmunity and joint inflammation, and it underscores why nutrition is increasingly considered a component of comprehensive RA management [24,127]. Table 1 summarizes key dietary components associated with modulation of gut microbial composition and immune-related pathways relevant to RA, highlighting that anti-inflammatory dietary patterns are linked to beneficial microbial taxa and immunoregulatory responses, whereas Western-style diets are associated with pro-inflammatory dysbiosis and higher disease activity.

### 4.4. Limitations of Current Nutritional and Microbiome Evidence

The interpretation of existing literature regarding nutrition, the microbiome, and RA is constrained by several factors. Nutritional assessments in these studies often rely on subjective self-reporting, introducing recall bias and measurement error. Furthermore, many observational studies are susceptible to reverse causation, where RA-induced changes in dietary habits, rather than diet itself, drive observed microbial shifts. Additionally, confounding factors in these studies, such as variations in medication exposure (specifically DMARDs and biologics), recent antibiotic use, and underlying batch effects in sequencing technologies, can significantly influence microbiome composition and mask true dietary impacts [41].

## 5. Diet–Microbiome Interventions in RA: Clinical Evidence

Dietary interventions are increasingly evaluated in RA not only as symptomatic adjuncts, but as upstream modulators of mucosal immunity and microbial ecology that may influence systemic inflammation, cardiometabolic comorbidity, and therapeutic responsiveness. Contemporary trials typically assess disease activity indices (e.g., DAS28), patient-reported outcomes (pain, fatigue, function), inflammatory biomarkers (CRP, ESR, selected cytokines), and, less consistently, microbiome composition and microbial metabolites [171]. A recurring limitation in the field is the heterogeneity of baseline pharmacotherapy (DMARD class, dose stability), disease phase (remission/low activity vs. active RA), and intervention intensity (diet counseling vs. structured provision), which complicates attribution of clinical benefit to microbiome-mediated mechanisms. Nonetheless, several recent randomized and controlled studies support the clinical plausibility of diet-driven improvement in RA-relevant outcomes, while also highlighting the need for standardized microbiome endpoints and longer follow-up. Table 2 summarizes the key molecular mechanisms, associated clinical biomarkers, and corresponding dietary or therapeutic interventions discussed in this review, mapping the interactions within the oral–gut–immune–nutrition axis.

### 5.1. Nutritional Intervention Trials in RA: Recent Controlled Evidence

Among whole-diet interventions, Mediterranean-style dietary patterns have the most contemporary trial evidence, largely because they are feasible, scalable, and align with cardiovascular risk reduction priorities in RA. The MADEIRA randomized controlled trial tested a personalized isocaloric Mediterranean diet plan supported by a digital clinical decision support system alongside physical activity nudges versus usual care in women with RA in remission, reporting improvements in diet quality and patient-centered outcomes over 12 weeks, consistent with an anti-inflammatory lifestyle framework rather than isolated nutrient effects [172]. Similarly, the MEDRA randomized controlled trial compared a telehealth-delivered Mediterranean diet intervention against national healthy eating guidance over 12 weeks in adults with RA, demonstrating improvements in physical function and quality of life measures, while also suggesting that structured dietary support can produce measurable benefits even when participants are concurrently managed with standard medical therapy [139]. Although these studies were not designed as microbiome-first trials, Mediterranean patterns are mechanistically linked to increased intake of fermentable fibers and polyphenols, which can enrich SCFA-producing taxa and attenuate pro-inflammatory signaling; the key translational gap is that microbiome and metabolomic endpoints remain secondary or absent.

Beyond Mediterranean patterns, structured plant-forward strategies have re-entered modern RA trial designs with clearer safety monitoring and contemporary treat-to-target contexts. In an exploratory randomized trial, a short therapeutic fast followed by a plant-based diet was compared with standard dietary recommendations, with effects observed on disease activity and well-being outcomes, supporting the concept that rapid shifts in dietary exposures can induce clinically relevant changes in symptom burden and inflammatory milieu in at least a subset of patients [136]. However, fasting-based protocols are difficult to generalize, may not be appropriate for all patients (e.g., those with frailty, eating disorders, or certain metabolic comorbidities), and require careful medical supervision. Importantly, the mechanistic hypothesis in such trials increasingly points toward altered gut permeability, bile acid signaling, microbial substrate availability, and downstream immune recalibration, reinforcing the need for paired microbiome and metabolite measurements to establish causality [173,174].

Dietary trials that emphasize fiber enrichment offer a more direct bridge between nutrition, microbial metabolism, and immune modulation. A randomized human clinical trial of high-fiber multigrain supplementation over 12 weeks reported improvements in disease activity and circulating inflammatory/oxidative stress biomarkers, consistent with the hypothesis that increasing fermentable substrates can shift microbial metabolism toward anti-inflammatory SCFA profiles and improve systemic inflammatory tone [151]. While food-based fiber interventions have strong biological plausibility, trial designs still vary substantially in fiber dose, composition (soluble vs. insoluble), and background dietary context, which likely determine whether meaningful metabolite shifts occur.

A distinct but related clinical approach is ketogenic-adjacent metabolic modulation paired with fiber support. The MIKARA double-blind randomized controlled intervention examined medium-chain triglyceride (MCT)- induced ketosis combined with fiber in RA and reported effects on disease activity endpoints in the primary analysis framework, offering evidence that metabolic state (ketosis) and colonic substrate availability (fiber) may jointly shape immune outcomes [152]. This line of research is notable because it explicitly tests combined dietary “signals” that affect both host metabolism and microbial ecology, although external validity, adherence, and long-term safety require further evaluation.

Collectively, these recent trials support clinically observable improvements in RA-relevant outcomes from structured dietary interventions. Furthermore, anti-inflammatory dietary patterns also directly impact periodontal health, reducing the primary reservoir for systemic pathobionts. Recent evidence indicates that food-derived natural products, especially polyphenols and flavonoids, act as potent inhibitors of *P. gingivalis* virulence factors, specifically targeting gingipains (Rgp and KGP) to attenuate bacterial pathogenicity [175,176]. These compounds not only suppress bacterial growth and biofilm formation but also disrupt energy metabolism by interfering with membrane-bound carbohydrate-specific transferases [177,178]. Simultaneously, omega-3 fatty acid supplementation has been demonstrated to improve clinical parameters (probing pocket depth and clinical attachment level) when used as an adjunct to periodontal therapy [179]. This dual action (reducing the oral pathogen load and virulence while modulating the gut microbiome) reinforces the role of nutrition in intercepting the oral-gut axis at its source [180].

Nevertheless, the microbiome remains more often an implied mediator than a directly quantified, causally tested pathway in many diet trials. This gap is particularly important for a review centered on the oral–gut–immune–nutrition axis, because mechanistic inference requires alignment across diet exposure, microbiome change, metabolite change, and immune/clinical response within the same study.

### 5.2. Probiotics and Prebiotics: Supplementation Studies as “Microbiome-Directed” Adjuncts

In contrast to whole-diet interventions, probiotic and prebiotic trials are explicitly microbiome-directed and can be easier to standardize across participants. However, probiotic efficacy in RA depends on strain selection, viability, dosing, treatment duration, baseline microbiome state, and concurrent immunomodulatory therapy. Within the last five years, one notable randomized study reported that a mixture of probiotics reduced inflammatory biomarkers and improved oxidative/nitrosative stress profiles in people with RA, supporting adjunctive benefit on systemic inflammatory surrogates [181,182,183]. While biomarker improvements are encouraging, many probiotic trials remain limited by modest sample sizes, short duration, and variability in clinical endpoints, underscoring the need for multicenter replication with harmonized DAS28 and patient-reported outcome measures.

Prebiotic strategies, by contrast, aim to enhance endogenous beneficial taxa and metabolite production rather than introduce exogenous organisms. A recent randomized trial reported that inulin supplementation improved inflammatory indices, clinical outcomes, and quality of life in RA patients, positioning fermentable fiber as a practical adjunct with a plausible mechanism through increased SCFA production and improved barrier function [150]. Prebiotic trials are conceptually attractive in RA because they may promote butyrate- and propionate-producing pathways and reduce endotoxin-driven innate immune activation. However, interindividual variability in microbiome fermentative capacity means that responders and non-responders may differ markedly at baseline, again motivating stratification by baseline microbiome and metabolomic profiling.

Specifically, recent evidence indicates that while fiber generally supports anti-inflammatory pathways, it may paradoxically exacerbate arthritis in patients with high *P. copri* abundance by facilitating the overproduction of proinflammatory metabolites like succinate, rather than beneficial SCFAs [184]. Interestingly, this contrasts with findings in healthy individuals, where Prevotella-dominated microbiota efficiently ferment fiber into beneficial propionate [185], highlighting that the metabolic output of Prevotella is highly context-dependent on the host’s inflammatory state. This reinforces the need for microbiome-stratified trial designs to tailor dietary interventions to the individual’s gut microbiome composition to ensure beneficial rather than harmful effects.

Beyond gut modulation, prebiotics and probiotics may also exert direct effects on the oral microflora, potentially reducing the oral pathogen load [186,187]. Probiotic strains such as *Lactobacillus* and *Bifidobacterium* have shown the ability to compete with periodontal pathogens for binding sites and produce antimicrobial substances that inhibit *P. gingivalis* growth and biofilm formation in the oral cavity [188,189].

Recent systematic syntheses have begun to formalize the evidence base for diet, supplements, and probiotics in inflammatory arthritis. A 2025 systematic review evaluating specific diets, dietary supplements, and probiotics across RA and related conditions concluded that Mediterranean, vegan/plant-based, and anti-inflammatory diets can improve disease activity and quality of life measures in RA, while certain supplements demonstrate immunomodulatory effects; however, the review also emphasized heterogeneity and the limited number of high-quality trials for some interventions [190]. These contemporary syntheses support cautious integration of microbiome-directed supplementation into RA care as adjuncts, while reinforcing the need for more rigorous trials that incorporate microbiome endpoints and clinically meaningful thresholds of response.

### 5.3. Fecal Microbiota Transplantation: Evidence Status, Safety, and “Dietary Priming”

Fecal microbiota transplantation (FMT) represents the most direct strategy to remodel gut microbial communities, but its clinical use in RA remains investigational and requires robust safety validation. A registered clinical trial is evaluating encapsulated FMT from healthy donors in RA patients, focusing on its effects on gut bacterial composition and related outcomes under controlled conditions (ClinicalTrials.gov ID: NCT05790356). The existence of such trials reflects a broader shift toward testing microbiome replacement strategies in immune-mediated diseases, but robust, peer-reviewed randomized outcome data in RA are not yet established in the contemporary literature.

In parallel, safety and regulatory considerations are central, particularly because RA patients may use immunosuppressive therapies that can increase susceptibility to infection. A comprehensive 2024 review of FMT as an adjuvant therapy across diseases highlights both therapeutic promise and unresolved safety/regulatory challenges, including donor screening, product standardization, and risk of transmitting infectious or antimicrobial resistance determinants, issues that are especially relevant for immunomodulated populations [191]. For RA-focused translational framing, the key point is that FMT should not be treated as a uniform intervention: donor selection, delivery route, dosing frequency, recipient preparation, and concurrent diet can strongly influence engraftment and durability [192].

The concept of “dietary priming” is increasingly discussed in microbiome therapeutics: altering dietary substrate availability before and after microbiome transfer to facilitate engraftment of donor taxa and stabilize functional pathways (e.g., SCFA production) [193]. Although RA-specific randomized evidence on dietary priming is not yet definitive, the logic is mechanistically coherent: if the recipient diet remains low in fermentable substrates and high in emulsifiers/ultra-processed foods, selective pressures may resist engraftment of beneficial anaerobes. Thus, future RA trials are likely to integrate standardized pre-FMT diets (often fiber-enriched, polyphenol-rich patterns) to improve engraftment and reduce volatility in microbial communities. Importantly, this approach aligns with the broader oral–gut–immune–nutrition axis: the intervention is not only microbial replacement but also ecological engineering through sustained dietary exposures.

### 5.4. Synergistic Effects: Diet + Microbiome Modulation + DMARDs

The most clinically consequential question is whether diet- and microbiome-targeted strategies can improve RA outcomes when added to disease-modifying antirheumatic drugs (DMARDs), either by enhancing response rates, reducing residual symptoms in remission/low disease activity, or enabling safer de-escalation strategies. Recent evidence supports the concept that baseline microbiome state relates to pharmacologic response. A study of new-onset RA found that the pretreatment gut microbiome was associated with lack of response to oral methotrexate (MTX), suggesting that microbial composition and/or function may influence drug metabolism, host immune tone, or mucosal permeability in ways that affect clinical efficacy [11]. This finding strengthens the argument for combined therapeutic strategies: if the microbiome contributes to DMARD non-response, then microbiome modulation could plausibly shift a patient toward responsiveness, although this remains to be proven in prospective interventional designs.

Diet trials conducted in medically treated RA cohorts also provide indirect support for synergy. Both MEDRA and MADEIRA were implemented in populations receiving contemporary medical management, implying that dietary intervention can incrementally improve functional and quality of life outcomes, even when pharmacologic inflammation control has been achieved [1,2]. This matters clinically because residual pain, fatigue, and impaired function persist in a subset of patients despite low inflammatory activity, potentially reflecting altered nociceptive processing, metabolic inflammation, microbiome-driven immune activation, or comorbid conditions.

Preclinical evidence further supports combined strategies. A 2025 experimental study reported that a symbiotic formulation (including a defined microbial component and an omega-3 fatty acid lysine salt) enhanced the therapeutic efficacy of low-dose tofacitinib in murine arthritis, outperforming either component alone in measured disease parameters [12]. While animal models cannot be directly extrapolated to human RA, the study illustrates a biologically plausible framework: targeted microbial/metabolic modulation may permit lower effective doses of immunomodulators or improve therapeutic indices. In human translation, this would require rigorous safety monitoring, particularly given infection risks with JAK inhibitors and the theoretical risk of introducing live organisms in immunosuppressed hosts.

## 6. Future Directions and Personalized Nutrition

RA remains a clinically heterogeneous disease in which patients differ substantially in symptom trajectories, comorbidity burden, and therapeutic response. This heterogeneity has sharpened interest in precision strategies that can (i) stratify patients by molecular endotypes, (ii) identify modifiable drivers of inflammation, and (iii) deliver interventions tailored to the patient’s biology and context. Multi-omics studies in RA increasingly support a systems view in which immune activation, metabolic reprogramming, and host-microbe interactions co-evolve across the course of disease, including preclinical stages and early treatment windows. Recent syntheses of RA multi-omics emphasize that integrating genomics, transcriptomics, proteomics, metabolomics, and microbiome profiling can reveal convergent pathways and candidate biomarkers that may inform individualized management, rather than relying on single-layer associations that often fail to generalize across cohorts [194].

Within this framework, the microbiome is attractive as both a biomarker source and a therapeutic target because it is responsive to diet, medications, and lifestyle. Microbiome-guided nutrition in RA aims to move beyond “one-size-fits-all” dietary advice by tailoring recommendations to microbial features linked to inflammation, barrier integrity, and immunometabolic signaling. However, the field is still transitioning from associative signatures to reproducible, clinically actionable models. Recent observational and mechanistic work illustrates the feasibility of this direction: baseline gut community structure and functional capacity have been linked to differential response to methotrexate (MTX), suggesting that pretreatment microbiome states may help anticipate non-response and guide earlier escalation or adjunctive strategies [195]. In parallel, large real-world cohort analyses in DMARD-naive RA indicate that microbiome status can predict short-term clinical improvement after DMARD initiation, while DMARDs themselves appear to shift dysbiotic patterns toward a more “eubiotic” state, highlighting bidirectional host-drug-microbiome dynamics that future nutrition trials must account for [196].

A central future direction is the construction of RA “endotypes” that combine microbiome, metabolome, immune phenotyping, and clinical features into interpretable, mechanistically grounded subgroups. Multi-omics reviews in RA underscore that immune signaling and synovial inflammation are tightly coupled to metabolic pathways, and that metabolomic and microbial readouts may provide proximal indicators of inflammatory tone and treatment responsiveness [194]. However, the practical challenge is not simply generating more data; it is harmonizing sample processing, controlling confounding (diet, drugs, geography, comorbidities), and producing models that generalize across populations.

Methodological roadmaps from nutrigenomics and precision nutrition are increasingly relevant to RA. Triangulation approaches (integrating nutrigenomics, metabolomics, and microbiomics) propose analytic strategies to connect genetic susceptibility and host metabolism with microbially derived metabolites and diet-responsive pathways [197]. In RA, this implies designing pipelines that map dietary exposures to microbial functions (not only taxa), then to metabolite patterns (e.g., SCFAs, bile acid derivatives, tryptophan metabolites), and finally to immune phenotypes such as Th17/Treg balance, cytokine networks, and autoantibody profiles. Such integrative architectures are likely to outperform taxonomic biomarkers alone because RA-associated microbial shifts often differ by ethnicity, medication exposure, and disease stage, whereas functional outputs may converge on shared immunometabolic pathways [198,199].

To translate endotypes into interventions, future studies will need to operationalize “actionable features”. Examples include low abundance of SCFA producers, high proteolytic fermentation potential, enrichment of pro-inflammatory pathobiont functions, or metabolomic patterns consistent with impaired barrier resilience [200]. These features can be paired with dietary prescriptions designed to shift microbial metabolism (e.g., fiber quality and diversity, fermented foods, polyphenol-rich patterns) while simultaneously aligning with cardiometabolic risk reduction, critical in RA, where cardiovascular comorbidity is common [201]. Furthermore, unified dietary strategies can target the oral-gut axis concurrently. Specifically, anti-inflammatory, polyphenol-rich patterns can disrupt the gingipain-dependent virulence of *P. gingivalis* in the oral cavity [175,177] while reducing proteolytic fermentation and increasing the production of beneficial metabolites in the gut [185,189].

Precision nutrition in RA will depend heavily on measurement quality. Traditional dietary assessment (food frequency questionnaires, recalls) is limited by recall bias and poor temporal resolution, particularly in fluctuating diseases where symptoms, medication changes, and diet adherence vary week-to-week [202]. Digital health platforms are increasingly positioned to close this gap by combining app-based food logging, barcode scanning, photographic meal capture, and linkage to wearables (sleep/activity), potentially alongside point-in-time symptom tracking and medication adherence. A 2025 review of personalized nutrition in the digital health era highlights how integrating real-time data streams can enable dynamic dietary adjustment and monitoring, while also emphasizing persistent barriers, including privacy, cost, and clinical validation [203].

Although most digital nutrition evidence is cardiometabolic rather than RA-specific, it is mechanistically relevant because the same platforms can capture high-frequency exposures that shape microbial ecology. A randomized trial of a personalized nutrition program incorporating individual metabolic responses and microbiome data demonstrated measurable improvements in cardiometabolic markers and microbiome beta-diversity, supporting the plausibility of app-delivered, data-driven dietary personalization at scale [204]. RA trials can adapt these architectures but must incorporate disease-specific endpoints (DAS28/CDAI trajectories, flare frequency, pain and fatigue scores, inflammatory markers, and ideally immune and metabolite readouts) to establish clinical value beyond general wellness outcomes.

As datasets become multimodal (diet, microbiome, metabolome, clinical labs, imaging, patient-reported outcomes), AI approaches are likely to become essential for pattern detection, feature selection, and individualized prediction. A 2025 scoping review of AI for precision nutrition describes rapid expansion since 2020 and highlights common gaps that are directly relevant to RA translation: limited external validation, inconsistent evaluation metrics, underrepresentation of minority populations, and insufficient attention to cultural dietary context [205]. These issues are not technical footnotes; they determine whether microbiome-guided nutrition will benefit diverse RA populations or amplify disparities.

In RA specifically, microbiome-based prediction has already been demonstrated in treatment response contexts. In new-onset, drug-naive RA, baseline gut metagenomics supported machine-learning prediction of MTX non-response and suggested functional links to MTX metabolism [195]. More recent observational work in DMARD-naive cohorts extends this concept beyond a single drug and suggests that stool and saliva microbiomes can contribute to response prediction after DMARD initiation [196]. These studies take a next step: integrating diet data and microbial metabolite profiles into clinically deployable decision-support tools that recommend nutrition strategies as adjuncts, particularly for patients at high risk of poor response, intolerance, or persistent inflammation.

However, RA-focused decision support must be designed around clinical workflows. A realistic near-term objective is not fully automated dietary prescriptions, but risk stratification and structured suggestions aligned with existing clinical counseling (e.g., identifying patients likely to have low SCFA output and recommending feasible fiber diversification; flagging patterns consistent with high inflammation and low diet quality; tracking adherence and symptom correlations) [206]. A longer-term objective is closed-loop personalization in which microbiome and metabolite monitoring guide iterative dietary adjustments, potentially synchronized with medication optimization.

To optimize therapeutic selection, future decision-support tools must incorporate specific molecular and clinical markers: the *Prevotella*/*Bacteroides* ratio in the gut should be analyzed to guide personalized nutritional interventions, such as high-fiber diets in *Prevotella*-dominated profiles [184,185]. Furthermore, routine assessment for periodontal disease is crucial, as periodontal treatment significantly reduces *P. gingivalis* load and improves clinical RA outcomes, specifically lowering disease activity scores (DAS28), particularly in patients with high baseline bacterial burden and antibody responses [207].

For microbiome-guided nutrition to be publishable, reproducible, and clinically credible, data governance and standardization must mature in parallel with biological discovery. A 2025 practical guide on FAIR data management for multi-omics and AI emphasizes that multimodal biomedical data frequently sit in silos and that interoperability is increasingly critical as AI use expands; it outlines pragmatic approaches to metadata capture and data sharing consistent with major funder policies [208]. For RA nutrition studies, this translates into standardized reporting of diet measurement methods, sequencing pipelines, batch effects, medication exposures (including antibiotics, PPIs, steroids), and clinical endpoints, along with transparent model documentation (training/validation splits, external cohorts, calibration).

Privacy is particularly salient when linking diet logs (behavioral data), purchasing patterns, and health data. A 2025 expert perspective on personalized nutrition data fusion emphasizes the need for cross-disciplinary standards, privacy-preserving data sharing, and representative datasets to ensure equitable access and validity [209]. These principles are directly applicable to RA, where socioeconomic status and food access strongly influence diet feasibility, and where digital interventions may inadvertently exclude patients with limited connectivity or health literacy.

Even if mechanistically sound, personalized nutrition will fail clinically if it cannot be implemented in routine care. Implementation challenges cluster into several domains.

Clinical feasibility depends on time, staffing, and reimbursement. Rheumatology clinics rarely have embedded nutrition specialists, and dietary counseling is often brief. Digital tools may extend reach, but they must be integrated with electronic health records and clinical workflows in ways that minimize burden [210,211]. Patient adherence is constrained by pain, fatigue, mood, work schedules, and food environments; therefore, personalization must include behavioral and socioeconomic context, not only omics.

Equity is a defining issue. AI-driven precision nutrition reviews stress the importance of culturally appropriate models and datasets that include underserved populations [205,211]. If RA microbiome datasets remain dominated by high-income settings, predictions may generalize poorly, and diet recommendations may be unrealistic or culturally incongruent. This risk is amplified by known geographic variability in microbiome composition driven by diet, sanitation, and antibiotic exposure. Equity-focused trial recruitment, multilingual digital interfaces, and food-plan flexibility should be considered core scientific design features, not add-ons.

Safety and clinical boundaries must also be explicit. RA patients may be immunosuppressed, and dietary supplements or probiotic products are variably regulated. Personalized recommendations should prioritize whole-diet patterns with clear safety profiles and define contraindications (e.g., infection risk, gastrointestinal comorbidities, pregnancy, and renal impairment). Importantly, personalized nutrition should be framed as adjunctive to DMARDs rather than a substitute, with a goal of improving inflammatory control, comorbidity risk, and quality of life.

## 7. Conclusions

The accumulated evidence synthesized in this review positions the microbiome–immune–nutrition axis as a central and biologically coherent framework for understanding RA pathogenesis and progression. Alterations in the oral and gut microbiota influence mucosal barrier integrity, innate and adaptive immune activation, and the generation of pathogenic autoantibody responses, while dietary patterns act as upstream modulators of microbial composition and metabolic output. Rather than a single causal microorganism or pathway, RA emerges as the consequence of a dynamic, bidirectional network linking dysbiosis, immune dysregulation, and host metabolic-nutritional status.

Within this framework, diet-based strategies represent a particularly attractive therapeutic avenue. Anti-inflammatory dietary patterns, adequate intake of micronutrients such as zinc and vitamin D, enrichment of fermentable fibers that promote short-chain fatty acid production, and targeted use of prebiotics or probiotics may favorably reshape the microbiome and dampen inflammatory immune circuits. Importantly, current evidence suggests that such interventions are most relevant as adjuncts rather than alternatives to disease-modifying antirheumatic drugs, with the potential to enhance therapeutic response, mitigate systemic complications, and improve long-term safety profiles.

Despite substantial progress, translation into routine clinical practice remains limited by heterogeneity in study designs, interindividual variability in microbiome composition, and the predominance of cross-sectional or short-term trials. Future advances will depend on interdisciplinary research integrating rheumatology, immunology, microbiology, nutrition science, systems biology, and dentistry (or the study of oral inflammatory diseases such as periodontitis), alongside rigorously designed longitudinal and interventional studies. Such efforts are essential for defining causality, identifying responder subgroups, and ultimately enabling personalized, microbiome-informed nutritional strategies as part of comprehensive RA management.

## Figures and Tables

**Table 1:** Dietary components influencing gut microbiota composition and immune pathways relevant to RA.

Nutrient/Compound	Microbiota Effect	Immune Effect
Omega-3 fatty acids	Increased abundance of *Lactobacillus* spp.; enhanced short-chain fatty acid production [160]	Reduced TNF-α and interleukin-6; modulation of eicosanoid pathways [158]
Fermentable fibers	Enrichment of butyrate-producing bacteria [144]	Expansion of Treg; suppression of Th17-mediated inflammation; improved epithelial barrier function [149]
Polyphenols (e.g., EGCG)	Reduced Proteobacteria; increased *Bifidobacterium* and *Akkermansia* [170]	Inhibition of nuclear factor κB signaling; antioxidant and immunomodulatory effects [130]
Vitamin D	Increased microbial diversity; enrichment of commensal taxa [165]	Suppression of interleukin-17; enhancement of mucosal immune tolerance and barrier integrity [161]
Zinc	Indirect support of microbial stability via epithelial barrier integrity and antioxidant defense [166]	Supports innate and adaptive immunity, maintains epithelial barriers, and provides antioxidant defense in RA [168]
Western diet (high fat/sugar)	Reduced *Faecalibacterium*; enrichment of pro-inflammatory taxa such as *Prevotella* [44]	Increased systemic inflammation; impaired gut barrier function; amplification of pro-inflammatory cytokine signaling [131]

**Table 2:** Summary of the oral–gut–immune–nutrition axis.

Axis/Component	Molecular Mechanism	Clinical Biomarker
Oral (Periodontal)	Citrullination by bacterial PAD (*P. gingivalis*) and NLRP3 inflammasome activation by toxins (*A. actinomycetemcomitans*).	Anti-citrullinated protein antibodies (ACPAs), Rheumatoid Factor (RF), subgingival bacterial load.
Gut (Intestinal)	Dysbiosis (reduced diversity, increased *P. copri*), decreased SCFA production, and increased intestinal permeability.	Fecal/serum SCFA levels (butyrate, acetate), fecal calprotectin.
Systemic Immune	Th17/Treg imbalance, systemic production of pro-inflammatory cytokines (IL-6, TNF-α, IL-1β).	Serum cytokines (IL-6, TNF-α), DAS28 (Disease Activity Score).

## Data Availability

No new data were created or analyzed in this study. Data sharing is not applicable to this article.
